# Understanding dynamics of pandemics

**DOI:** 10.3906/sag-2004-133

**Published:** 2020-04-21

**Authors:** Levent AKIN, Mustafa Gökhan GÖZEL

**Affiliations:** 1 Department of Public Health, Faculty of Medicine, Hacettepe University, Ankara Turkey; 2 Department of Infectious Diseases, General Directorate of Public Health, Ministry of Health, Ankara Turkey

**Keywords:** Pandemic, new coronavirus infection, COVID-19

## Abstract

Along the centuries, novel strain of virus such as influenza produces pandemics which increase illness, death and disruption in the countries. Spanish flu in 1918, Asian flu in 1957, Hong Kong flu in 1968 and swine flu in 2009 were known pandemic which had various characteristics in terms of morbidity and mortality. A current pandemic is caused by novel corona virus originated from China. COVID-19 pandemic is very similar to Spanish, Hong Kong, Asian and swine influenza pandemics in terms of spreading to world by the mobilized people. Burden of pandemic is considered in terms of disease transmissibility and the growth rate of epidemic and duration of pandemic can be calculated by transmissibility characteristic. The case definition, finding out cases and first case cluster, proper treatment, sufficient stockpiles of medicine and population cooperation with the containment strategy should be considered for reduction of burden of pandemic.

## 1. Introduction

Pandemics, are well-known as the epidemics on the basis of worldwide spread and cause excessive number of sickness, deaths in the world and cause disruption for social economic situation of the countries which were affected. As the result of the globalization, change of lifestyles, social and economic improvement caused the emerging infection and accelerated occurrence and circulation of new microbial agents beside this globalization has also facilitated sharing information and experiences. 

Many pandemics have occurred throughout the history of mankind. Primarily plague, smallpox, cholera and Spanish flu are the longest-lasting, repetitive, and caused large numbers of human deaths. In the beginning of 20th century, at least 20 million people deaths were estimated due to1918 Spanish flu. The first originated place of the pandemic is not clear, but many experts assumed that the possible origin was in China. However, the first outbreaks occurred at the same time in North America in March 1918 [1]. 

Influenza spread to across the US country. Infection was spread to France in April 1918 by US naval forces. From France, infection transmitted to British Military Forces in May 1918, and spread to Italy, Spain and Germany at the same month. After that North Africa India, China, New Zealand and the Philippines were suffered from pandemic flu, infection expanded to every country in a short time. At the end of the First World War, Spanish flu caused many more deaths than all the troops. The pandemic has widespread to all continents with the mobilization of the military troops. Although pandemics in mankind history spread mostly along trade and communication lines, the spread of the 1918 Spanish flu was transmitted from region to region through military mobilization and trench warfare, limited health services poor sanitation made easy transmission [2].

In course of 1918-1919 influenza pandemic, adults were affected mostly when compared with children and older people [3]. According to mortality statistics of US, the mortality rate of people aged 20–34 and pregnant women were higher than mortality of the older than 50 years of age. This pattern is also occurred in other influenza pandemics^1^1Institute of Medicine of the National Academies (2005). The threat of pandemic influenza: are we ready? Workshop Summary [online]. Website https://www.nap.edu/catalog/11150/the-threat-of-pandemic-influenza-are-we-ready-workshop-summary [accessed 08 April 2020].. 

## 2. Dynamics of pandemics

Spanish flu pandemic is reported to spread in three waves: first wave in spring 1918 was moderate but rapidly spread, second wave in autumn 1918 was completely severe and destructive and third wave in spring 1919 was more severe than the first wave but not worse than second wave^2^2Institute of Medicine of the National Academies (2005). The threat of pandemic influenza: are we ready? chapter 1: the story of influenza [online]. Website https://www.ncbi.nlm.nih.gov/books/NBK22156/pdf/Bookshelf_NBK22156.pdf [accessed 8 April 2020].. Many countries experienced the second and third waves of more virulent form of infection. It is estimated that 50% of the world’s population have infected, 25% manifested clinical signs [4]. After almost forty years of Spanish flu, a new influenza strain was detected in China. The 1957 pandemic was caused by A H2N2 strain [5]. The virus spread to Hong Kong, Singapore, Taiwan, and Japan and in summer spread to world in the summer of 1957. Two main routes of spread were: firstly, across Russia to Scandinavia and Eastern Europe, and secondly from the US to other countries. Asian flu pandemic had spread to the world in 6 months. The pandemic affected approximately 40 ± 50% of people, and 25 ± 30% of them had clinical signs. Most of the deaths were due to secondary bacterial pneumonia. Mortality rate was calculated as 1 per 4000. The mortality was high among children and elderly people [1]. The 1957 (Asian) influenza pandemic, estimated the basic reproduction number (R0) was 1.8 and 60%–65% of infected individuals were manifested clinical symptoms [6].

Excess mortality due to Spanish flu was 598 deaths per 100,000 people per year. However excess mortality of Asian flu was only 40.6% [7].

After a decade occurrence of Asian flu pandemic, the new flu strain H3N2 was caused a new pandemic known as the Hong Kong flu in 1968. Although new virus was extremely transmissible, degree of severity was milder than the Asian flu. Excess mortality of Hong Kong flu was 16.9%. Virus was spread through Vietnam War veterans returning to the United States. Then Hong Kong flu was seen in Japan, England, Wales, Australia, and Canada in 1969 [8,9]. At this pandemic the characteristic mortality shift towards younger populations, the case fatality rate was highest among children [10]. Hong Kong flu is estimated to have caused between 500,000 and two million deaths worldwide in two waves [11]. The burden of pandemic was higher in countries with increases in excess all-cause mortality of 9.1%–13.0%, than in US. Impact of pandemic was present the geographic heterogeneity [8]. Hospitalization was significantly high amongthe elderly. Hong Kong flu pandemic showed that public health intervention strategies and medical science were insufficient and not improved between the 1957 and 1968 pandemics [12].

In April 2009, a new pandemic occurred that H1N1pdm09 virus was causative agent, emerged from Mexico. Within few weeks, the disease had spread across many countries. The global trade and travel served swine flu to spread as 122 countries in six weeks, however past pandemics had spread in six months [13]. H1N1 pandemic profile had a course with three waves in spring, summer, and fall. The pattern was generally mild wave in the spring and early summer, but reemerged more severe after opening the schools. Studies showed that adults older than 50 years of age were less susceptible to 2009 H1N1 infection than younger adults. While compared the previous pandemics, the infectivity was higher among children than among adults (Table) [14].

**Table 1:** Baseline characteristics of different pandemics.

Pandemic(years andcommon name)	Reference	Causativeagent	Area Of emergence	Estimated reproductive number	Secondary attack rates (%)	Estimated case-fatality rate	Age groups most affected
1918–1919(Spanish flu)	[6]	Influenza A (H1N1)	Unclear	1.7–2.8	-	NA	Adult, pregnant
1957–1958(Asian flu)	[8]	Influenza A (H2N2)	Southern Chine	1.8	18.5–26.8	NA	Children, elderly
1968–1969(Hong Kong flu)	[11]	Influenza A (H3N2)	Southern Chine	1.06–2.06	15.0	NA	Elderly
2009(swine flu)	[18]	Influenza A (H1N1)	Mexico	1.4–1.6	17,5	NA	Adolescent, young adults
2019–(COVID-19)		A Novel Coronavirus(SARS-CoV-2)	Wuhan, Chine	5.7	< 2	2.3	Elderly

NA: Not applicable.

Household studies explained the serial interval of the disease. Serial interval estimates are provided data in spreads speed to inform recommendations on the period that patients should stay home, and to estimate the effect in treatment on transmission [15]. The R0 for 2009 Pandemic Influenza A (H1N1) has been estimated to be between 1.4 and 1.6 [16]. Secondary attack rates in pandemic showed significant variation, i.e. estimates of 33% in children and for adults 22% in different regions. Case fatality rates were obtained from high-income countries and varied three to nine times among the studies. According to one study, people younger than 64 years accounted for 80% of these deaths. Southeast Asia and Africa had highest mortality among of the other continent [17]. After three influenza pandemics in 20thcentury, it is expected when the first pandemic will be seen in 21st century. Humankind did not encounter a new influenza pandemics but a novel corona virus pandemic (Figure). As in pandemics in history, its spread to all over the world through people traveling between countries in two-three months. In the past, pandemics spread somewhat predictably along military passages or important trade routes, but globalization has multiplied and obscured the dominant routes. Given their sheer number, and the speed with which human and animal transmission vectors can move, there is no longer a single dominant pathway for the geographical movement and expansion of infectious diseases. Pandemics are inherently uncertain, considering experiences and preparations that we have gained from past pandemics, the epidemiologic characteristics of pandemic should be evaluated. By the way the impact of the control measures are be able to evaluate during the COVID-19 pandemic’s days.

**Figure 1:**
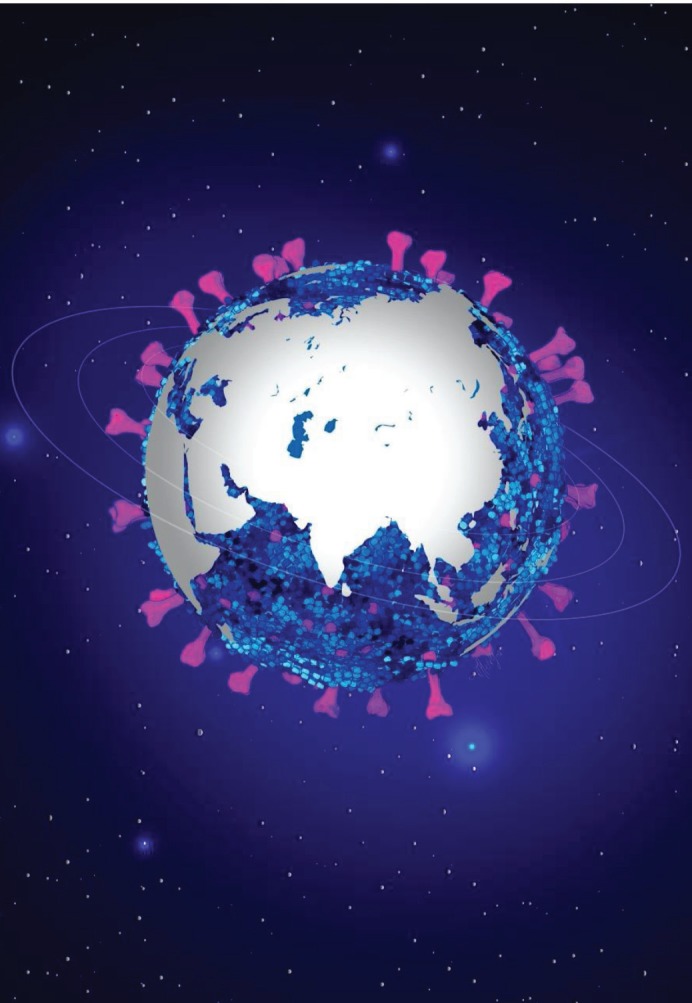
A pandemic is a threat for all globe (Source: Courtesy of Merve EVREN, PhD, Ege University).

## 3. Discussion

When a COVID-19 was identified, the information was obtained in various studies that epidemiological and clinical evidence or clues were needed to indicate when and what type precautions should be implemented. In general, acute respiratory infections are the leading, particularly affecting the youngest and oldest people in developing countries. These types of infections are caused by viruses or mixed viral–bacterial infections and spread fast. The most of the primary mode of transmission of acute respiratory diseases is transmitted via droplets, sometimes transmission occur through contact (including hand contamination followed by self-inoculation) or infectious respiratory aerosols at short distance^3^3 World Health Organization (2014). Infection prevention and control of epidemic- and pandemic-prone acute respiratory infections in healthcare [online]. Website https://apps.who.int/iris/bitstream/handle/10665/112656/9789241507134_eng.pdf?sequence=1 [accessed 8 April 2020]..

The distribution and the outcome of epidemic varies according to several factors such as household crowding, humidity, temperature, season, hygiene, access to health-care facilities, and isolation capacity, demographic characteristics of population, cigarette-smoking, host ability to transmit infection, immune status, nutritional status, prevalence of comorbidity diseases, characteristics of pathogenic, modes of transmission, transmissibility, virulence factors, etc. 

COVID-19 transmitted mainly through person to person contact via respiratory droplet by coughing and sneezing. Most of the cases were 30 to 79 years of age and the proportion was found 87%, under 19 years and 80 years or older were respectively 2% and 3% [18]. The proportion of asymptomaticpeople which was testing positive for SARS-CoV-2, varied between 5% and 80%^4^4Centre for Evidence-Based Medicine (2020). COVID-19: what proportion are asymptomatic? [online]. Website https://www.cebm.net/covid-19/covid-19-what-proportion-are-asymptomatic/ [accessed 8 April 2020].. In China, all clusters investigated and 78%–85% were within families and secondary attack rate in household was found 3%–10%^5^5World HealthOrganization (2020). Report of the WHO-China joint mission on coronavirus disease 2019 (COVID-19) [online]. Website https://www.who.int/docs/default-source/coronaviruse/who-china-joint-mission-on-covid-19-final-report.pdf [accessed 8 April 2020].. Similar study was carried out in the US and a symptomatic secondary attack rate for all household contacts among all contacts was found 10.5% [19]. Household contacts and travelling with an infected person increased the risk (OR:6-7). The household secondary attack rate was 15%, and the observed serial interval mean was 6.3 days. The median incubation period was 4.8 days (95% CI 4.2–5.4). Symptoms were manifested within 14.0 days (95% CI 12.2–15.9) of infection^6^6Bi Q, Wu Y, Mei S, Ye C, Zou X et al. (2020). Epidemiology and transmission of COVID-19 in Shenzhen China: analysis of 391 cases and 1,286 of their close contacts [online]. Website https://www.medrxiv.org/content/10.1101/2020.03.03.20028423v3.article-info [accessed 8 April 2020].. R0 was likely estimated to be 5.7 ( 95% CI 3.8–8.9) in the US [20].

The overall case-fatality rate 2.3% and this rate was 8.0% for aged 70–79 years and 14.8% for 80 years and older. Case-fatality rate was higher and varied between 5.6% and 10.5% [18]. 

COVID-19 pandemic is very similar to Spanish, Hong Kong, Asian and swine influenza pandemics in terms of spreading to world. Although countries were suffered from pandemic, the epidemiologic dynamic varied from country to country, from pandemic to pandemic. The common features of pandemics are spread to all over the world through human mobilization (such as military troops, national or international travel for different purpose etc.) and severity increasing for person with chronic diseases. 

## 4. Conclusion

Burden of pandemic is considered in terms of disease transmissibility and the rate of epidemic grows, time of peaks, expected how many people to be affected, and duration of pandemic can be calculated by transmissibility characteristic. Hospitalization and fatality are used as the indicators of the severity. At the beginning of a pandemic, the characteristic of pandemic agent is unknown. Therefore, surveillance method and analyzing data should be planned in advance [21]. Consequently, determination of the first case cluster, case definition and finding out cases, effective treatment to the targeted population, sufficient stockpiles of medicine and population cooperation with the containment strategy and, in particular, any social distance measures introduced should be considered for reduction of burden of pandemic. 

## Acknowledgments/disclaimers

Levent AKIN is a member of COVID-19 Advisory Committee of Ministry of Health of Turkey.

Mustafa Gökhan GÖZEL is a member of COVID-19 Advisory Committee of Ministry of Health of Turkey.
